# Age : pleural fluid ADA ratio and other indicators for differentiating between tubercular and malignant pleural effusions

**DOI:** 10.1097/MD.0000000000029788

**Published:** 2022-06-30

**Authors:** Jiupeng Zhou, Yuanli Yang, Yongfeng Zhang, Heng Liu, Quanli Dou

**Affiliations:** Xi’an Chest Hospital, Xi’an 710000, Shaanxi Province, China

**Keywords:** age/ADA ratio, Cancer Ratio, Cancer Ratio plus, pleural effusion

## Abstract

Most of pleural effusions are caused by tuberculosis and malignant tumor. Difficult sampling and bacterial sparing nature of these diseases challenge doctors’ diagnosis in China.

This study aimed to develop a new convenient and effective method for the differentiation of tuberculous and malignant pleural effusion.

A prospective cohort study of patients hospitalized with malignant (n = 90) and tuberculous (n = 130) pleural effusions from September 2018 to October 2020 was performed. The diagnostic performance of the age to pleural fluid ADA ratio (age/ADA) and other indicators to distinguish tuberculous and malignant pleural effusions was evaluated by receiver operating characteristic (ROC) curve analysis.

The areas under the curve (AUC) of age/ADA and pleural fluid ADA were largest. Age/ADA showed sensitivity and specificity of 81.5% (95%CI 73.8%–87.8%) and 97.8% (95%CI 92.2%–99.7%) respectively. The sensitivity and specificity of pleural fluid ADA were 83.1% (95%CI 75.5%–89.1%) and 93.3% (95%CI 86.1%–97.5%) respectively. The positive likelihood [36.69 (95%CI 9.3–144.8)] of age/ADA was significantly higher than that of pleural fluid ADA [12.46 (95%CI 5.7–27.1)]. The AUCs for Cancer Ratio and Cancer Ratio plus were lower and showed a sensitivity of 80.0% (95%CI 72.1%–86.5%), 80.0% (95%CI 70.2%–87.7%) and a specificity of 81.5% (95%CI 73.8%–87.8%), 80.0% (95%CI 70.2%–87.7%) respectively.

Age/ADA has a higher diagnostic accuracy than ADA. Age/ADA is a promising diagnostic index for tuberculous and malignant pleural effusion with high sensitivity and specificity, especially the high positive likelihood ratio. The diagnostic accuracy of Cancer Ratio and Cancer Ratio plus are inferior to those of age/ADA and ADA.

## 1. Introduction

Pleural effusion (PE) is common in the emergency department, respiratory department, or chest diseases.^[1]^ Tuberculous pleural effusion (TPE), malignant pleural effusion (MPE), and parapneumonic pleural effusion are the most common cause of clinical exudative pleural effusion.^[2]^ Difficult sampling and bacterial sparing nature of these diseases challenge doctors’ diagnosis. Especially, TPE and MPE are the most difficult to be distinguished.

For the past few years, some more advanced tests have been designed to identify the origin of PE. A meta-analysis including 20 studies indicated that the overall estimates of the sensitivity/specificity for differentiating MPE were as below: CEA+CA125, 0.65/0.98, CEA+CA15-3, 0.64/0.98, CEA+CA19-9, 0.58/0.98, CEA+CYFRA21-1, 0.82/0.92, and CA15-3+CYFRA21-1, 0.88/0.94.^[3]^ Although the detection of tumor markers has certain potential, their sensitivity and specificity are not satisfactory. Moreover, they are often costly and lack of operability. The summary sensitivity and specificity of Xpert MTB/RIF were 30% (95%CI: 21%–42%) and 99% (95%CI: 97%–100%) for diagnosing tuberculous pleurisy in Zhen-Yu Huo’s meta-analysis.^[4]^ This method of combining a pleural lactate dehydrogenase (LDH) to adenosine deaminase (ADA) ratio and a pleural CEA level had a sensitivity of 62.0% and a specificity of 91.0%.^[5]^ Similarly, sensitivity was not satisfactory.

The initial treatment decision, based on the changes of biochemical markers, such as LDH, ADA level and lymphocyte percentage in PE, is critical. So, Piotr claimed lately that age to pleural fluid ADA ratio (age/ADA) was characterized by high sensitivity (93.2%) and fair specificity (71.2%) for differentiating MPE from non–MPE.^[6]^ Verma reported serum LDH: pleural fluid ADA ratio (Cancer Ratio) showed sensitivity and specificity of 0.98 (95%CI 0.92–0.99) and 0.94 (95%CI 0.83–0.98) at a cut-off level of >20.^[7]^ His other study showed that the sensitivity and specificity of Cancer Ratio: pleural fluid lymphocyte count (Cancer Ratio Plus), a cut-off level of >30 were 97.6% (95% CI 0.90–0.99) and 94.1% (95% CI 0.78–0.98).^[8]^ These biochemical indicators are encouraging, but majority of these studies are retrospective and small sample. In addition, others’ reports about these indicators are rare.

Consider the above factors, our purpose is forward-looking to assess the effectiveness of age/ADA in differentiating TPE and MPE, and evaluate the ablility of “Cancer Ratio” and “Cancer Ratio Plus” in differentiating TPE and MPE.

## 2. Methods

### 2.1. Data collection

This study is a study of diagnostic accuracy. We prospectively enrolled patients with PE hospitalized from September 2018 to October 2020. Patients who participated in the study either showed signs of PE when they first from patients who participated in the study eithershowed signs of PE when they first went to Xi’an Chest Hospital, or had PE during hospitalizationundergoingchest CT scanvisited to Xi’an Chest Hospital, or had pleural effusion during hospitalization undergoing chest CT scan. Patients who had transudative effusion and parapneumonic pleural effusion (PPE) were removed. All patents involved in this study gave their written informed consent. Minors (<18 years old) were not included in the study. The study obtained approval from the Ethics Committee of Xi’an Chest Hospital. MPE was diagnosed by malignant pleural fluid cytology or malignant pleural biopsy histology. TPE was defined by growth of mycobacterium tuberculosis on pleural fluid, including polymerase chain reaction or epithelioid granuloma in pleural biopsy tissue. The pathological results were judged by 2 professional pathologists. Tuberculosis in PE was detected by 2 professional laboratory physicians. The pathologists and laboratory physicians were blind to the results of the other test and to any other clinical information.

To estimate the group size, we calculated the sample size according to the sensitivity and specificity of age/ADA reported by Piotr. With α = 0.05, 2-tailed and a power of 90%, we needed 25 patients in TPE group and 79 patients in MPE group. Considering a compliance rate of 90 %, we asked 28 TPE patients and 88 MPE patients to participate in this study.

These parameters from patients who participated in the study either showed signs of PE when they first went to Xi’an Chest Hospital, or had pleural effusion during hospitalization undergoing chest CT scan were assessed, including patient gender, age, final diagnosis, fever, abnormal lump (lump > 1 cm revealed physical or iconography examination), C-reactive protein, serum lactate dehydrogenase (LDH), and pleural fluid biochemical parameters (LDH, ADA, pleural fluid lymphocyte count).

### 2.2. Three ratios were defined

1. The ratio of age and PE ADA (age/ADA): this ratio to assess this accuracy of combining age with PE ADA to identify TPE and MPE.
2. Cancer Ratio was described as serum LDH: pleura fluid ADA ratio, which was a predictive factor of tuberculous pleural effusion.
3. The ratio of Cancer Ratio to pleural fluid lymphocyte count was called as Cancer Ratio plus. This is calculated to be prospective to evaluate the effectiveness of differentiating TPE and MPE.

### 2.3. Statistical analysis

Statistical analysis was carried out by MedCalc 18.0 (MedCalc Software, Ostende, Belgium) software package. The data were described by median and quartile in skew distribution and mean and standard deviation in normal distribution. The normality of a distribution for the continuous variables was assessed by Kolmogorov-Smirnov test. The Kruskal-Walis test was used for the difference of continuous variables. Differences of categorical variables were analyzed by Chi square test. A multivariate logistic regression analysis was used to analyze the variables with difference by kruskal-Walis test or chi square test. The diagnostic performance of the variables associated with TPE shown by multivariate logistic regression analysis to distinguish TPE and MPE were evaluated by receiver operating characteristic (ROC) curve, consisting of area under the curve (AUC) and 95% confidence interval (CIs). ROC curves were compared by Delong test, which represented the diagnostic performance of different tests. *P* < 0.05 was considered statistical significance.

## 3. Results

A total of 261 patients were recruited who either showed signs of PE when they first visited to Xi’an Chest Hospital, or had PE during hospitalization. Three patients younger than 18 years old were excluded. Twenty-three patients were removed who had transudative effusion (n = 16) and PPE (n = 7). A total of 235 patients was tested in the study. In index test negative group (age/ADA > 2.65, n = 118), 5 patients were not diagnosed because 2 patients died before diagnosis and 3 patients refused to undergo pathological or etiological tests. One patient was not confirmed by pathology or etiology. In index test positive group (age/ADA ≤ 2.65, n = 117), 7 patients were not diagnosed because 3 patients died before diagnosis and 4 patients refused to undergo pathological or etiological tests. Two patients were not confirmed by pathology or etiology. Finally, a total of 220 patients was included in the study.

There were 90 patients with MPEs (40.9%), 130 patients with TPEs (59.1%). The primary causes of MPE included: primary lung cancer (n = 68), pleural metastatic carcinoma of unknown origin (n = 8), mesothelioma (n = 6), gastric carcinoma (n = 4), carcinoma of urinary bladder (n = 2), and lymphoma (n = 2). General clinical features and selected biochemical parameters of patients with MPE and TPE were shown in Table 1. There were only conventional symptomatic treatment including xygen inhalation, nutritional support, etc, without any special treatment, such as antituberculosis, antiinfection or antitumor treatment between the index tests and reference standard. There were 1 case of hemothorax and 2 cases of pneumothorax from performing the index test or the reference standard.

**Table 1:** General clinical features and selected biochemical parameters in patients.

Variable	Total (N = 220)	MPE (N = 90)	TPE (N = 130)	*P* value
Age	56(32–69)	64(56–75)	39(24.75–62.75)	<0.001
Gender (male/female)	142/78	52/38	90/40	0.081
More than 5 years smoking history	102/118	36/54	66/64	0.115
Fever (yes/no)	70/150	10/80	60/70	<0.001
Abnormal lump	81/139	66/24	15/115	<0.001
Pleural fluid ADA	22.5(9–42)	9(5–12.25)	38(25.5–51)	<0.001
Pleural fluid LDH	687.83 ± 458.91.	672.42 ± 334.37	724.66 ± 523.11	0.076
Serum LDH	516.15 ± 415.71	555.38 ± 436.39	459.48 ± 379.10	0.093
C-reactive protein	29.34(9.54–55.96)	20.56(3.33–39.35)	41.25(13.61–80.68)	0.001
Age/ADA	2.83(0.83–7.22)	6.89(4.80–11.21)	1.06(0.58–1.97)	<0.001
Cancer ratio	19.16(10.96–38.33)	40.62(30.08–62.66)	12.51(8.95–17.52)	<0.001
Pleural fluid lymphocyte count	0.80(0.78–0.80)	0.80(0.76–0.80)	0.80(0.78–0.80)	0.229
Cancer ratio plus	26.28(13.35–54.11)	54.22(37.15–81.33)	15.99(11.29–22.21)	<0.001

Univariate analysis showed that there was not remarkable difference in gender, more than 5 years smoking history, pleural fluid LDH, serum LDH and pleural fluid lymphocyte count between the 2 groups. Pleural fluid ADA and C-reactive protein increased in tuberculous pleural effusion. In addition, the age of malignant PE group was significantly older and the fever rate was significantly lower. When combined with age, serum LDH, pleural fluid ADA and pleural fluid lymphocyte count, there were greater differences between the 2 groups (Table 1).

We further evaluated the independent influences on TPE of the parameters with difference shown by univariate analysis. In multivariate logistic regression analysis, Fever (OR = 58.695, *P* = 0.013) and pleural fluid ADA (OR = 1.276, *P* < 0.001) were positive predictive factors of tuberculous pleural effusion. In contrast, Age/pleural fluid ADA (OR = 0.456, *P* < 0.001), Cancer Ratio (OR = 0.880, *P* = 0.025), Cancer Ratio plus (OR = 0.902, *P* = 0.025) and abnormal lump (OR = 0.020, *P* = 0.001) maintained significance as negative predictive factors of tuberculous pleural effusion. C-reactive protein (OR = 1.089, *P* = 0.433) or age (OR = 0.998, *P* = 0.921) did not predict the origin of pleural effusion (Table 2).

**Table 2:** Multivariate logistic regression analysis with TPE as the outcome variable.

Variable	Coefficient	Standard error	Exp (B)	95%Cl		*P* value
				lower	Upper	
Age	−0.002	0.025	0.998	0.951	1.047	0.921
Fever	4.072	1.636	58.695	2.375	1450.591	0.013
Pleural fluid ADA	0.243	0.057	1.276	1.142	1.425	0.000
C-reactive protein	0.041	0.017	1.089	0.928	0.993	0.433
Age/ADA	−0.784	0.209	0.456	0.303	0.688	0.000
Cancer ratio	−0.127	0.057	0.880	0.787	0.984	0.025
Cancer ratio plus	−0.103	0.046	0.902	0.825	0.987	0.025
Abnormal lump	−3.891	1.208	48.981	4.590	522.628	0.001

ROC analysis was performed to obtain the cut-off level in order to get the best balance between sensitivity and specificity for these 6 variables (fever, pleural fluid ADA, Age/pleural fluid ADA, Cancer Ratio, Cancer Ratio plus and abnormal lump) those were significantly associated with TPE in the multivariable logistic regression analysis.

The 2 indexes of the largest AUC were age/ADA and pleural fluid ADA, showing a high sensitivity and specificity for differentiating TPEs and MPEs. The AUCs for Cancer Ratio and Cancer Ratio plus were lower. The AUCs for fever and abnormal lump plus were the lowest.

**Figure 1.**
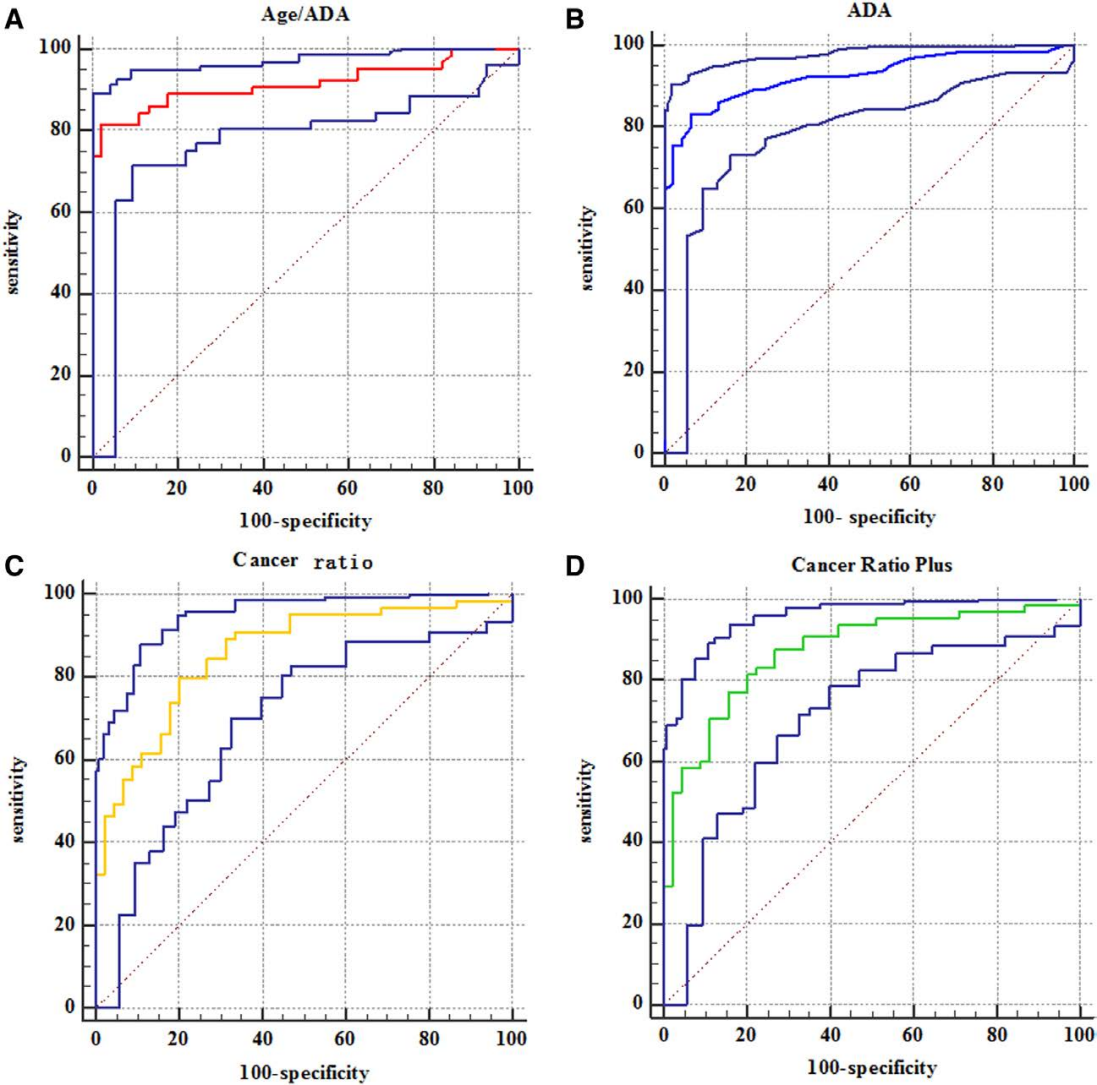
Receiver operating characteristic (ROC) curve for age/ADA (A), ADA (B), Cancer Ratio (C), Cancer Ratio plus (D). The middle curve represents the ROC curve. The top and bottom curves represent 95% CI.

The sensitivity and specificity of age/ADA were 81.5% (95%CI 73.8%–87.8%) and 97.8% (95%CI 92.2%–99.7%) respectively at a cut-off level of ≤2.65. The positive likelihood ratio (PLR) was 36.69 (95%CI 9.3–144.8), and the negative likelihood ratio (NLR) was 0.19 (95%CI 0.1–0.3). The AUC was 0.916. The sensitivity and specificity of pleural fluid ADA were 83.1% (95%CI 75.5%–89.1%) and 93.3% (95%CI 86.1%–97.5%) respectively at cut-off level of >21.5. The PLR was 12.46 (95%CI 5.7–27.1), and the NLR was 0.18 (95%CI 0.1–0.3). The AUC was 0.925. For Cancer Ratio, the sensitivity and specificity were 80.0% (95%CI 72.1%–86.5%) and 80.0% (95%CI 70.2–87.7%) at cut-off level of ≤21.24, respectively. The PLR was 4.00 (95%CI 2.6–6.1), and the NLR was 0.25 (95%CI 0.2–0.4). The AUC was 0.859. The sensitivity and specificity of Cancer Ratio plus were found to be 81.5% (95%CI 73.8%–87.8%) and 80.0% (95%CI 70.2%–87.7%) respectively at the cut-off level of ≤34.74. The PLR was 4.08 (95%CI 2.7–6.2), and the NLR was 0.23 (95%CI 0.2–0.3). The AUC was 0.874. The sensitivity and specificity of fever were found to be 46.1% (95%CI 37.4%–55.1%) and 88.8% (95%CI 80.5%–94.5%) respectively at the cut-off level of >37.3°C. The PLR was 4.15 (95%CI 2.2–7.7), and the NLR was 0.61 (95%CI 0.5–0.7). The AUC was 0.675. The sensitivity and specificity of abnormal lump were found to be 87.7% (95%CI 80.8%–92.8%) and 73.3% (95%CI 63.0%–82.1%) respectively at the cut-off level of lump ≥ 1cm. The PLR was 3.29 (95%CI 2.3–4.7), and the NLR was 0.17 (95%CI 0.1–0.3). The AUC was 0.805 (Table 3, Fig. 1). There were not indeterminate or missing data during the index tests.

**Table 3:** Comparison of different parameters differentiating between TPE and MPE.

Variable	AUC	95% CI	Cut-off value	Sensitivity (95%CI), %	Specificity (95%CI), %	+LR (95% CI)	–LR (95% CI)
Fever	0.675	0.609–0.737	>37.3	46.1(37.4–55.1)	88.9(80.5–94.5)	4.15(2.2–7.7)	0.61(0.5–0.7)
Pleural fluid ADA	0.925	0.882–0.956	>21.5	83.1(75.5–89.1)	93.3(86.1–97.5)	12.46(5.7–27.1)	0.18(0.1–0.3)
Age/ADA	0.916	0.871–0.949	≤2.65	81.5(73.8–87.8)	97.8(92.2–99.7)	36.69(9.3–144.8)	0.19(0.1–0.3)
Cancer ratio	0.859	0.806–0.903	≤21.24	80.0(72.1–86.5)	80.0(70.2–87.7)	4.00(2.6–6.1)	0.25(0.2–0.4)
Cancer ratio plus	0.874	0.822–0.914	≤34.74	81.5(73.8–87.8)	80.0(70.2–87.7)	4.08(2.7–6.2)	0.23(0.2–0.3)
Abnormal lump	0.805	0.747–0.855	≥1cm	87.7(80.8–92.8)	73.3(63.0–82.1)	3.29(2.3–4.7)	0.17(0.1–0.3)

## 4. Discussion

PE is a common extrapulmonary tuberculosis worldwide and is also the commonest form of tuberculosis.^[9]^ Most of PEs are caused by tuberculosis and malignant tumor.^[10]^ The differentiation of 2 types of pleural effusion is a challenge for clinicians. Therefore, it is necessary to develop a new convenient and effective method for the diagnosis of pleural effusion.^[11]^

Previous literature reported that the morbidities of great majority of malignant tumors increased with age. For example, most of the malignant tumors reported from 2011 to 2013 occurred in patients aged 60 or older in Poland and increased with age, and the highest incidence was between 80 and 90 years old.^[12]^ In Britain, 50% of lung carcinoma patients are over 70 years old.^[13]^ A large-scale epidemiological study conducted by Zhang et al indicated that most young people aged 15 to 24 suffered from pleural tuberculosis.^[14]^ In multivariate logistic regression analysis, we found that age was not significant as a negative predictor of tuberculous pleural effusion. This may be related to the inclusion of older tuberculous pleural effusion patients. However, when combined with age and ADA, a cut-off level of age/ADA ≤ 2.65 was highly predictive of tuberculous pleural effusion in patients, with both high sensitivity 81.5% (95%CI 73.8–87.8%) and specificity 97.8% (95%CI 92.2–99.7%). The PLR was 36.69 (95%CI 9.3–144.8), while the NLR was 0.19 (95%CI 0.1–0.3). These were slightly different from the results reported by Piotr. He thought that the sensitivity and specificity of age to pleural fluid ADA was 93.2% and 71.2% respectively for differentiating MPE from nonMPE.^[6]^ The difference may be due to the sensitivity and specificity in Piotr’s study based on MPE. The sensitivity and specificity of pleural fluid ADA were 83.1% (95%CI 75.5–89.1%) and 93.3% (95%CI 86.1–97.5%) respectively at cut-off level of >21.5. A meta-analysis also showed that the sensitivity and specificity of ADA in the diagnosis of tuberculosis pleural effusion were 0.93 and 0.90, respectively.^[15]^ ADA is an enzyme in lymphocytes and myeloid cells. It is indispensable for DNA metabolism and cytoactive. It can recycle the poisonous purine way of metabolites. ADA levels are usually ascended in inflammatory effusions, such as pleural, pericardial and articular effusions caused by bacterial infection and granulomatous inflammation, as well as malignant tumors and autoimmune diseases.^[16,17]^ Though the AUC of pleural fluid, ADA was slightly larger compared with age/ADA (Z = 0.346, *P* = 0.7297, Fig. 1A, B), the PLR of age/ADA was 36.69 and was considerably higher than that of pleural fluid ADA. This meant that patients were 36.69 times more likely to have tuberculous pleural effusion than not when age/ADA ≤ 2.65. This probability was high enough to suggest that the pleural effusion would be likely to be tuberculous. This suggested that age/ADA was more advantageous in differentiating tuberculous and malignant pleural effusion than ADA alone. There was no significant difference in the negative predictive value between age/ADA and pleural fluid ADA.

We found that the sensitivity and specificity of Cancer Ratio were 80.0% (95%CI 72.1%–86.5%) and 80.0% (95%CI 70.2%–87.7%) respectively at cut-off level of ≤21.24 and the AUC was 0.859 (Fig. 1C), which were significantly lower than the pooled sensitivity (0.97) and specificity (0.89) of Cancer Ratio shown in the meta-analysis of Yan Qiu Han.^[18]^ The results were also significantly different from what were reported by Verma^[8]^ and Piotr.^[6]^ The sensitivity and specificity of Cancer Ratio plus were found to be 81.5% (95%CI 73.8%–87.8%) and 80.0% (95%CI 70.2%–87.7%) respectively at the cut-off level of ≤34.74 in our study (Fig. 1D). Similarly, this result was obviously inferior to that in Verma’s previous research.^[7]^ This difference might be related to different inclusion standard and the different features of the research objects. One reason was that the benign PE group was different. The proportions of patients with TPE and PPE were 26% and 21% in Piotr’s study.^[6]^ TPE constituted only 28.8% in the study by Verma.^[8]^ His other study included 40 patients with tubercular effusion, 14 with parapneumonic effusion, and 9 with undiagnosed (24.5%, 8.5%, and 5.5%).^[7]^ In our study, patients with tuberculous PE accounted for 59.1%. The other reason was that the proportion of lung cancer in malignant PE group was different. Lung cancer accounted for 95% and 97.6% of malignant PE respectively in both studies of Verme.^[7,8]^ In Piotr’s study, malignant PE contained 51.4% of lung cancer patients.^[6]^ The proportions of patients with lung cancer were 75.6% in our study. Serum lactate dehydrogenase (LDH) is an extensive cellular enzyme that increases in a nonspecific manner in response to tissue damage. It was found that serum LDH was increased in many clinical cases.^[19]^ We also found that serum LDH was higher in patients with malignant PE, which was consistent with previous reports.^[7,20]^ The level of serum LDH may be related to the type and metastasis of tumor, and the inclusion of more advanced cancer cases and different types of patients may lead to different sensitivity and specificity.

In addition, though fever was a positive predictor of tuberculous PE, while abnormal lump was negative, the sensitivity and specificity of the both methods were poor in differentiating tuberculous and malignant PE.

The present study had 2 limitations. First, we did not study exudative effusion caused by other diseases, such as parapneumonic effusion, to verify the outcomes in this group of patients. Second, lung cancer accounted for a large proportion in patients with malignant PE.

All in all, our study shows that age/ADA has a higher diagnostic accuracy than ADA. Age/ADA is a promising diagnostic index for tuberculous and malignant PE with high sensitivity and specificity, especially the high positive likelihood ratio. The diagnostic accuracy of Cancer Ratio and Cancer Ratio plus are inferior to those of age/ADA and ADA. However, as a result of the limitations of our study, further studies need to be carried out to demonstrate our results.

### Author contributions

Conceptualization: Jiupeng Zhou, Quanli Dou; Data curation: Jiupeng Zhou; Funding acquisition: Jiupeng Zhou, Quanli Dou; Investigation: Yuanli Yang, Yongfeng Zhang; Methodology: Jiupeng Zhou, Heng Liu; Project administration: Quanli Dou; Software: Jiupeng Zhou; Roles/Writing – original draft: Jiupeng Zhou; Writing – review & editing: Quanli Dou.
